# Roles of Long Noncoding RNAs in Recurrence and Metastasis of Radiotherapy-Resistant Cancer Stem Cells

**DOI:** 10.3390/ijms18091903

**Published:** 2017-09-05

**Authors:** Hsiang-Cheng Chi, Chung-Ying Tsai, Ming-Ming Tsai, Chau-Ting Yeh, Kwang-Huei Lin

**Affiliations:** 1 Radiation Biology Research Center, Institute for Radiological Research, Chang Gung University/Chang Gung Memorial Hospital, Linkou, Taoyuan 333, Taiwan; hgchi@mail.cgu.edu.tw; 2 Kidney Research Center and Department of Nephrology, Chang Gung Immunology Consortium, Chang Gung Memorial Hospital, Chang Gung University College of Medicine, Taoyuan 333, Taiwan; monster_0616@yahoo.com.tw; 3 Department of Nursing, Chang-Gung University of Science and Technology, Taoyuan 333, Taiwan; mmtsai@mail.cgust.edu.tw; 4 Department of General Surgery, Chang Gung Memorial Hospital, Chiayi 613, Taiwan; 5 Liver Research Center, Chang Gung Memorial Hospital, Linkou, Taoyuan 333, Taiwan; chautingy@gmail.com; 6 Department of Biochemistry, College of Medicine, Chang-Gung University, Taoyuan 333, Taiwan; 7 Research Center for Chinese Herbal Medicine, College of Human Ecology, Chang Gung University of Science and Technology, Taoyuan 333, Taiwan

**Keywords:** radiotherapy, radioresistance, CSCs, EMT, metastasis, LncRNAs

## Abstract

Radiotherapy is a well-established therapeutic regimen applied to treat at least half of all cancer patients worldwide. Radioresistance of cancers or failure to treat certain tumor types with radiation is associated with enhanced local invasion, metastasis and poor prognosis. Elucidation of the biological characteristics underlying radioresistance is therefore critical to ensure the development of effective strategies to resolve this issue, which remains an urgent medical problem. Cancer stem cells (CSCs) comprise a small population of tumor cells that constitute the origin of most cancer cell types. CSCs are virtually resistant to radiotherapy, and consequently contribute to recurrence and disease progression. Metastasis is an increasing problem in resistance to cancer radiotherapy and closely associated with the morbidity and mortality rates of several cancer types. Accumulating evidence has demonstrated that radiation induces epithelial–mesenchymal transition (EMT) accompanied by increased cancer recurrence, metastasis and CSC generation. CSCs are believed to serve as the basis of metastasis. Previous studies indicate that CSCs contribute to the generation of metastasis, either in a direct or indirect manner. Moreover, the heterogeneity of CSCs may be responsible for organ specificity and considerable complexity of metastases. Long noncoding RNAs (lncRNAs) are a class of noncoding molecules over 200 nucleotides in length involved in the initiation and progression of several cancer types. Recently, lncRNAs have attracted considerable attention as novel critical regulators of cancer progression and metastasis. In the current review, we have discussed lncRNA-mediated regulation of CSCs following radiotherapy, their association with tumor metastasis and significance in radioresistance of cancer.

## 1. Introduction

Radiotherapy has remained one of the mainstay treatments for cancer in the clinic for over 100 years. The principle of the treatment is based on the theory that cancerous regions are more sensitive than normal tissues to radiation because cancer cells have a limited ability to repair damaged DNA and tend to divide more quickly, while the normal tissue parts surrounding tumor lesions can withstand radiotherapy and recover [1]. However, the biological complexity and heterogeneity of cancers lead to certain tumor types being more resistant to radiotherapy. Importantly, resistance to treatment often leads to subsequent recurrence and metastasis of cancer in numerous patients [2,3]. Previous studies have reported that intrinsic cancer stem cells (CSC) representing a small subpopulation of cancer cells existing within heterogeneous tumors are responsible for radioresistance and metastasis in various cancer types [4,5,6]. In contrast, rather than CSCs, the progeny that differentiates from CSCs accounting for substantial tumor regions is hypothesized to be sensitive to radiotherapy, leading to short-term regression of cancer. Failure to treat and prevent cancer is therefore attributable to the fact that radiotherapy is aimed at the tumor bulk but not CSCs [7]. Findings to date have implied that radiation can paradoxically enhance tumor recurrence and metastasis [3,8,9].

The process of cancer metastasis is thought to consist of several steps. The initial escape from the primary region requires the epithelial tumor cells to become motile and degrade the underlying basement membrane and extracellular matrix (ECM). Activation of epithelial–mesenchymal transition (EMT) is considered necessary to allow epithelial cancer cells local invasion and dissemination at distant organs [10,11]. Moreover, radiation induces EMT in several cancer cell types [12,13]. EMT is closely linked to CSC generation and radioresistance [2,14,15]. As mentioned above, several CSC characteristics are relevant to metastasis, such as motility, invasiveness, and resistance to radiotherapy.

Therefore, regulation of CSCs and therapies that specifically target stem cells are required for prevention of radiation-induced metastasis and developing improved radiotherapeutic strategies. A small fraction of the human genome (~1.5%) codes for proteins [16]. The majority of the remaining noncoding regulatory regions transcribed are defined as noncoding RNAs (ncRNA). ncRNAs have been shown to influence a variety of human diseases, including cancers. Long noncoding RNAs (lncRNAs) are a subclass of ncRNAs implicated in the development and progression of cancers [17]. Several investigations on large clinical cancer samples have demonstrated that specific lncRNAs, such as HOX transcript antisense RNA (*HOTAIR*) and Growth arrest specific 5 (*GAS5*), can influence the outcomes of radiotherapy and act as valuable prognostic biomarkers [18]. Increasing studies have focused on the biological functions and mechanisms of lncRNAs in recurrence or progression following radiotherapy.

While the relationship between lncRNAs and CSCs has gradually become an important topic in cancer research, the specific cellular mechanisms by which these RNAs regulated in the cancer stem cells and subsequently affect recurrence and metastasis of radioresistant cancers remain unclear at present. In the current review, we have summarized recent studies focusing on: (1) the relationship between CSCs and radiotherapy; (2) underlying mechanisms implicated in radiation-induced metastasis, radioresistance and CSC generation; (3) roles of lncRNAs participating in radioresistance and radiotherapy-induced cancer metastasis; and (4) roles of lncRNAs in the progression and metastasis of CSCs. It is speculated that the long noncoding RNAs potentially contribute to radioresistant tumor occurrence and metastasis by affecting the population or behavior of cancer stem cells. Elucidation of the molecular cues underlying the effects of noncoding RNAs on CSCs may thus facilitate the design of effective strategies to improve radiotherapy and prevent cancer metastasis.

## 2. Cancer Stem Cells and Radiotherapy

Several reports to date have demonstrated that CSCs serve as the crucial contributor to radioresistance and recurrence after radiotherapy in the majority of cancers, including lung cancer, breast cancer and hepatocellular carcinoma (HCC) [4,5,6,19]. CSCs are defined as a small population of cancer cells within tumors that exhibit self-renewal capacity. These cells can effectively differentiate into the heterogeneous lineages of tumor cells constituting a specific cancer type [20]. At present, it is hypothesized that tumor development is triggered by the capacity of self-renewal and multi-lineage differentiation of CSCs, whereas differentiated offspring of CSCs do not display the ability to self-renew and extensively proliferate, therefore losing tumorigenesis potential [21].

CSCs were initially identified in human acute myeloid leukemia (AML) with the capacity to reconstruct the original disease entirely over several transplantations into immunocompromised mice. In this study, self-renewal and differentiation properties were only detected within the immature CD34^+^/CD38^−^ population of cells [22]. CSCs from solid tumors were identified initially in breast tumors [23] and subsequently in a broad spectrum of solid tumors, including colorectal, brain, melanoma, pancreatic, ovarian, lung, prostate and gastric cancers [24]. As CSCs display similar characteristics as normal stem cells with self-renewal and differentiation capacities, they have high clonogenic ability and can generate a serially transplantable phenocopy of the primary tumor in immunocompromised or syngenic animals [20]. At present, however, no reliable markers that allow precise measurement of CSCs in different tumors are available in the clinic. The most widely used strategy for isolation of CSCs is based on specific sets of surface markers [20]. Several specific surface markers of CSCs have been identified in diverse human tumors, including CD133, CD44, CD44^+^/CD24^−^ and CD34^+^/CD38^−^ [2,24]. Furthermore, specific membrane transporters and activities or expression patterns of enzymes in CSCs are different from those in non-CSCs. For instance, levels of adenosine triphosphate-binding cassette (ABC) transporters on the cell membrane are increased and consequently facilitate efflux of Hoechst dye in CSCs of several cancer types, including ovarian, lung, glioma and nasopharyngeal carcinoma [25,26,27]. ALDH1 (aldehyde dehydrogenase 1) activity in CSCs is additionally enhanced in several tumor types, such as lung, breast and pancreatic cancers [28,29,30]. Transcription factors, such as NANOG (Nanog homeobox), OCT4 (POU class 5 homeobox 1, POU5F1), SOX2 (SRY-box 2), c-MYC (MYC proto-oncogene, bHLH transcription factor) and KLF4 (Kruppel like factor 4), and signaling pathways, including WNT (Wingless-type MMTV integration site family), Hh (Hedgehog), Notch, TGF-β (Transforming growth factor beta), PDGF (Platelet derived growth factor) and JAK (Janus kinase 1)/STAT (signal transducer and activator of transcription), play crucial roles in maintaining self-renewal capacity in CSCs and therefore present potential targets in the development of therapeutic strategies [2,31]. In addition to surface markers and functional regulators, CSCs display unique characteristics, including increased levels of anti-apoptotic regulators, enhanced DNA repair efficiency and cellular quiescence [2,31].

The radioresistant ability of CSC markers/regulator-positive cells has been established [2,4,32,33,34]. Functional markers/regulators, together with the stem cell characteristics, influence the outcomes of radiotherapy [31,35,36]. Moreover, markers of CSCs may serve as predictors of clinical outcomes in patients receiving radiotherapy. In view of the crucial effects of CSCs on radiotherapy, clarification of the underlying mechanisms that mediate radioresistance is an urgent consideration. Generally, radioresistance of CSCs is associated with increased self-renewal capacity, activation of anti-apoptosis genes, enhanced capacity of DNA repair and reduced DNA damage via inhibition of reactive oxygen species (ROS) [36]. Additionally, radiotherapy has been shown to trigger EMT, and, consequently, metastasis and radioresistance of cancer cells [12,13]. The relationship between radiation and EMT is discussed below.

## 3. Mechanisms Implicated in Radiation-Induced Metastasis, Radioresistance and CSC Generation

### 3.1. Radiation Promotes Cancer Metastasis and Radioresistance via Induction of EMT

Metastasis is one of the major obstacles to successful cancer therapy [10,37,38] and closely associated with EMT, a biological process critical in embryogenesis, organ fibrosis and wound healing. Moreover, the EMT process confers mesenchymal phenotypes to epithelial cells, characterized by loss of epithelial phenotypes and markers, such as E-Cadherin, ZO-1 (tight junction protein 1, TJP1), and Desmoplakin, and simultaneous gain of mesenchymal markers, including N-cadherin, Vimentin, Fibronectin, and α-smooth muscle actin (α-SMA). Thus, cancer cells undergoing EMT gain invasive and metastatic abilities [10,11].

Notably, radiation is reported to trigger EMT and promote metastasis of several cancer types. For example, radiation treatment enhances the stability of β-catenin (catenin β 1) via the activation of PI3K (phosphatidylinositol-4,5-bisphosphate 3-kinase)/AKT (AKT Serine/threonine kinase), thereby inducing the expression and secretion of granulocyte colony-stimulating factor (G-CSF). Recent report also indicates that radiation promotes the expressions of Nrf2 and Notch1 to activate EMT process in non-small cell lung cancer (NSCLC) cells. Inhibition of Nrf2-Notch axis reduces EMT but enhanced radiosensitivity of NSCLC, and consequently decreases radiation-induced NSCLC invasion [39]. Another report also suggests that radiation treatment can activate TGF-β1 signaling to induce EMT process in Lewis lung carcinoma [40]. Additionally, irradiation induces EMT of human alveolar type II epithelial carcinoma A549 cells, characterized by elimination of E-cadherin and enhancement of Vimentin expression is mediated via TBK-GSK-3β axis [41].

In esophageal cancer cells, irradiation induces the EMT phenotype accompanied by increased migration, invasion, and radioresistance through the induction of SNAIL (Snail family transcriptional repressor 1), TWIST (Twist family bHLH transcription factor 1), IL-6 (interleukin-6) and STAT3 (signal transducer and activator of transcription 3) signals [42,43]. In glioma cells, sub-lethal doses of radiation have been shown to promote the metastatic ability through inducing the expression of α_v_β_3_ integrin, enhancing matrix metalloproteinase (MMP) activity, and altering the ratio of BCL-2 (B cell leukemia/lymphoma 2)/BAX (BCL2 associated X) toward the apoptosis-resistant phenotype [8,44]. Further, radiation treatment induces the expression and activity of MET though ATM (ATM serine/threonine kinase)-NF-κB (nuclear factor kappa-light-chain-enhancer of activated B cells) signaling pathway, and consequently promotes invasion and apoptosis-resistance of breast cancer cells [12]. Similar phenomena have been observed in nasopharyngeal carcinoma, colorectal, lung, and liver cancer subjected to radiation therapy [3,8,13,45,46]. Furthermore, preclinical and clinical evidence suggests that radiation enhances metastatic potential, in both the primary tumor region and normal tissues, under specific circumstances [3,47].

In addition to radiation-induced metastasis, cancer cells that have gained mesenchymal characteristics via EMT are more resistant to radiotherapy, indicating that radiation-induced EMT confers radioresistance, which contributes to relapse [48]. For example, prostate cancer cells have been shown to exhibit EMT phenotypes and become more resistant to radiation after radiotherapy [49]. Similar results have been reported with other cancer types, including nasopharyngeal carcinoma, colorectal cancer (CRC), lung cancer, HCC cells, breast cancer and gastric cancer [45,50,51,52,53,54].

The process of EMT contributes to radioresistance of cancer cells via induction of EMT-related genes/signals, such as PI3K/AKT, PTEN (phosphatase and tensin homolog), mTOR (mechanistic target of rapamycin kinase) and TGF-β, which inhibit cell death signals. Accordingly, silencing the expression of SNAIL and SLUG (Snail family transcriptional repressor 2), crucial EMT inducers, could sensitize cancer cells to genotoxic stress induced by chemo or radiotherapy [55]. In addition, activation of the Notch signaling pathway induces SNAIL and SLUG expression in tumor cells and the phenomenon of EMT, leading to the suppression of p53-mediated apoptosis induced by cancer therapy [14,56]. Therefore, initiation of EMT is accompanied by the activation of crucial gene sets or signals that influence sensitivity to radiotherapy.

**Figure 1:**
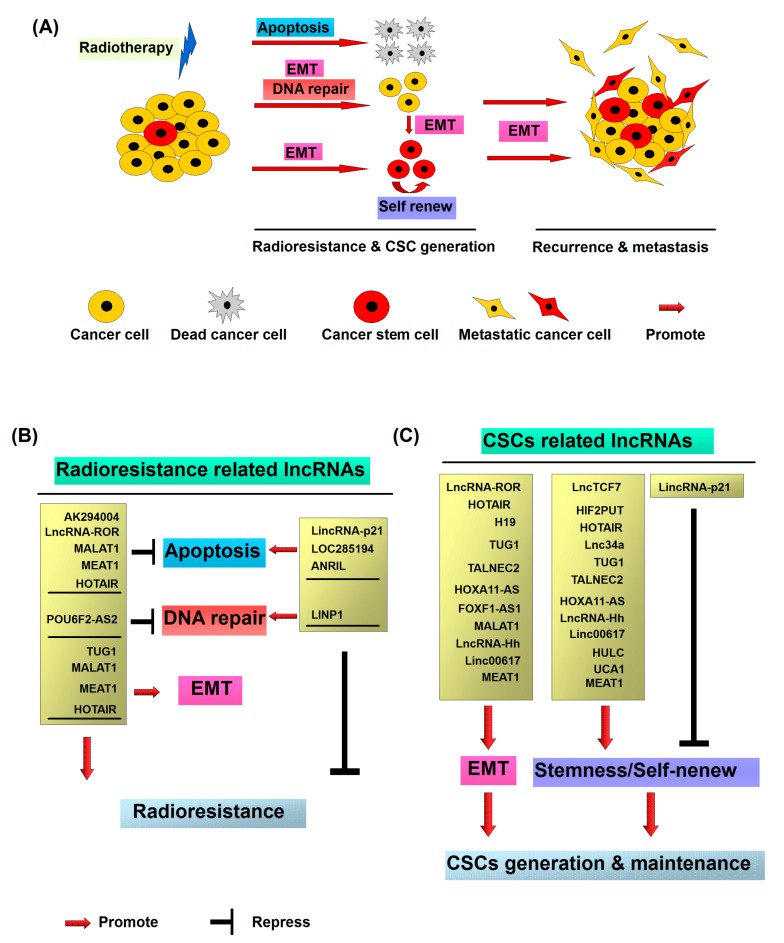
Molecular mechanisms of radiotherapy induced cancer recurrence and metastasis: (**A**) Cancer stem cells (CSCs) representing a small subpopulation of cancer cells existing within heterogeneous tumors are responsible for radioresistance and metastasis. After the radiation treatment, the majority of cancer cells are killed via the induction of apoptosis or mitotic death. However, a small number of non-CSCs exhibit the radioresistant property and dedifferentiate and transform into CSCs through radiation induced epithelial–mesenchymal transition (EMT). The newly generated CSCs from non-CSCs, together with the intrinsic CSCs, consequently contribute to recurrence and metastasis of cancer; (**B**) radioresistant CSC associated long noncoding RNAs (lncRNAs); and (**C**) CSC associated lncRNA.

As mentioned above, radiotherapy-induced EMT in cancer cells promotes the development of cancer cell radioresistance. Additionally, previous studies have demonstrated that EMT leads to the generation of CSCs generally resistant to therapy with elevated expression of genes involved in free-radical scavenging, DNA repair pathways and drug transporting capacity [57,58]. Interestingly, non-stem cancer cells can spontaneously dedifferentiate into CSCs via EMT. Thus, generation of CSCs after radiation via EMT may be an important factor in the development of resistance of cancer cells to radiotherapy. In conclusion, radiation treatment is prone to damage cancer cells and shrink tumors. However, irradiation triggers a small percentage of cancer cells to adopt the malignant phenotypes including stemness, metastasis and anti-apoptosis through EMT (Figure 1A). The inhibition of the signals associated with EMT represents a potentially efficient strategy for the treatment patients with radioresistant tumors, although further investigation is required.

### 3.2. Radiation Promotes CSC Generation

Radiation treatment causes enrichment of CSCs both in vitro and in vivo, implying that CSC generation is triggered by radiation. For instance, radiotherapy has been shown to enhance the population of CD44^+^ cells that display CSC properties in patients with prostate cancer [59]. Another study reported an increase in expression of CSC surface markers in the MDA-MB-231-xenograft model after stimulation with fractionated radiation [60]. Enrichment of breast CSCs via radiation is considered the result of different sensitivities of CSC and non-CSC cancer cells to radiotherapy. Notably, earlier studies have demonstrated that radiation promotes reprogramming of differentiated cancer cells into CSCs. Enrichment of breast CSCs after radiation stimulation is implicated in the induction of stem cell-like characteristics in non-stem cancer cells [61,62]. In these studies, ALDH1^−^ breast cancer cells in a single cell suspension were isolated from either human breast specimens or established cell lines and subsequently subjected to various doses of radiation. The number of ALDH1^+^ cells was dramatically increased in a dose-dependent manner after five days of radiation treatment. The results clearly indicate that radiotherapy can induce the CSC phenotype in non-stem breast cancer cells. Moreover, radiation-induced CSCs display better capacity of mammosphere formation and tumorigenicity, and express stem cell-related genes similar to breast CSCs isolated from samples without radiation treatment. In addition to breast cancers, radiotherapy could induce a stem cell-like phenotype in non-stem HCC cells [63]. Non-CSCs isolated from HepG2 and Huh7 cells display better sphere formation ability and express stem cell-related genes after exposure to radiation [63].

Non-stem cancer cells can generate cells with CSC properties via the EMT process [15,64,65]. Additionally, radiation treatment induces cancer cells to undergo EMT, leading to radioresistance [49]. For example, after radiotherapy, resistant cells display a complex phenotype involving a combination of the properties of CSCs and EMT with higher expression of Snail, CD44, CD24, and PDGFR-β (platelet derived growth factor receptor β) in non-small cell lung cancer (NSCLC) cells [6]. Additionally, the subpopulation of CD133^+^ colorectal or CD24^+^ ovarian cancer cells exhibits both properties of CSC and EMT, characterized as increased SNAIL, TWIST, and Vimentin along with decreased E-cadherin expression [66,67]. EMT is reported to induce transcriptional regulators or signaling pathways, such as SNAIL, STAT3, NF-κB, Notch and PI3K/AKT and the MAPK (mitogen-activated protein kinase) cascade, indicative of a critical role in radiation-induced CSC properties [2]. The collective findings suggest that radiation promotes the generation of CSCs from non-stem cancer cells and shed light on the novel interactions between cancer cells and radiotherapy, which pave the way for clarifying the precise mechanisms leading to cancer radioresistance.

## 4. Cellular Functions of LncRNAs in Radioresistance

With the advent of genome sequencing efforts [68,69], numerous RNA transcripts with similar properties to mRNAs that are not translated into proteins have been identified. These transcripts, collectively defined as long noncoding RNAs (lncRNAs), are generally primary non-protein coding sequences greater than 200 nucleotides in length [70]. Although the cellular function of the majority of lncRNAs is still unknown [70], a number of reports are suggested to be functional RNA molecules involved in the regulation of diverse biological processes [70]. LncRNAs can interact with DNA, RNA or proteins. Recent large-scale sequencing analyses have revealed that many transcripts of lncRNAs may, in fact, be translated into functional peptides [71]. Accumulating studies indicate that lncRNAs regulate the transcription of genes related to the DNA damage response via different regulatory modes, including signal, decoy, guide, and scaffold [72]. DNA damage response and repair capacity are closely related to sensitivity to radiotherapy. In addition, numerous lncRNAs are aberrantly expressed in cancer cells and have been implicated in development of the radioresistant phenotype of cancer cells. Thus, lncRNAs may present effective target molecules in combination with radiation treatment for cancer. Here, we have systematically reviewed documented literature focusing on the lncRNAs participating in resistance to radiotherapy.

### 4.1. LncRNAs Associated with Apoptosis

#### 4.1.1. *LincRNA-p21*

*LincRNA-p21* is an intergenic long noncoding RNA (3100 nucleotides) located on chromosome 17, ~15 kb upstream from the *Cdkn1a* (p21) gene [73]. *LincRNA-p21* has been identified as the downstream target of p53 modulating the expression of numerous genes involved in cell cycle control, DNA damage and repair pathways [73]. The RNA acts as a suppressor of p53-dependent transcriptional responses and its inhibition influences the expression patterns of genes that are generally repressed by p53. In the presence of DNA damage, *lincRNA-p21* is required to induce p53-dependent apoptosis via physical association with ribonucleoprotein K (hnRNP-K). This step leads to proper genomic localization of *lincRNA-p21*/hnRNP-K at the gene promoter region and consequently suppresses their expression in a p53-dependent manner [74]. Additionally, *lincRNA-p21* is implicated in cell cycle regulation. Specifically, *lincRNA-p21* is proposed to enforce the G1/S checkpoint and regulate cell proliferation via activating p21 expression in cis to promote Polycomb target genes expression [75]. Notably, expression of *lincRNA-p21* is downregulated in several cancer types, and recent reports have also demonstrated a role in radiation-mediated cell death [76,77]. *LincRNA-p21* is frequently reduced in colorectal cancer (CRC) cancer cell lines and human tissues and leads to elevation of the WNT/β-catenin signal pathway [77,78]. Furthermore, expression of *lincRNA-p21* is increased upon X-ray treatment. Higher levels of lincRNA enhance the sensitivity of CRC to radiotherapy via repression of β-catenin signals and induction of the proapoptotic gene, NOXA, consequently promoting apoptosis [77]. Silencing of *lincRNA-p21* causes β-catenin overexpression and leads to increased stemness and radioresistance of glioma stem cells [79]. Another study showed that *lincRNA-p21* is transcriptionally induced by ultraviolet B in a p53-dependent manner in keratinocytes in vitro or skin from mice in vivo. Ultraviolet B-mediated lincRNA-p21 triggered cell cycle arrest and apoptosis in keratinocytes, and conversely, its inhibition resulted in evasion of apoptosis caused by ultraviolet B [74].

#### 4.1.2. *LOC285194*

The lncRNA *LOC285194*, also termed LSAMP antisense RNA, was first identified as a p53-regulated tumor suppressor that influences the cell cycle and apoptosis by regulating VEGF receptor 1 and miR-211 in osteosarcoma [80,81]. Recent evidence has shown decreased expression of *LOC285194* in esophageal squamous cell carcinoma in relation to larger tumor size, high-grade TNM stage, lymph node and distant metastasis. Additionally, low expression of *LOC285194* serves as an independent prognosis factor closely associated with preoperative chemoradiotherapy response and poorer disease-free and overall survival rates [82]. Thus, *LOC285194* may be considered a potential therapeutic marker for screening of patients to determine their suitability for chemoradiotherapy and estimate outcomes.

#### 4.1.3. *ANRIL*

The lncRNA *ANRIL*, also designated *CDKN2B-AS* (CDKN2B antisense RNA 1), was initially identified from familial melanoma patients [83]. LncRNA *ANRIL* produces a 3834 nt RNA transcript in the antisense orientation of the *INK4B-ARF-INK4A* gene cluster. Previous studies have documented upregulation of ANRIL in various cancer types and its utility as a prognosis marker [84,85,86]. Upregulation of *ANRIL* in cancer cells has been shown to enhance resistance to radiotherapy via inhibition of apoptosis and induction of cell proliferation. Conversely, inhibition of *ANRIL* expression causes repression of cellular proliferation and radioresistance via induction of apoptosis. Further experiments revealed that oncogenic effects of *ANRIL* are mediated through negative regulation of miR-125a, a tumor suppressor implicated in apoptosis and metastasis [87]. Moreover, Silencing of ANRIL upregulates the expression of the pro-apoptotic genes, BAX and SMAC (second mitochondria-derived activator of caspases), but suppresses the anti-apoptotic gene, BCL-2 [88]. Thus, lncRNA *ANRIL* is considered an important suppressor of apoptosis that influences cancer cell sensitivity to radiotherapy.

#### 4.1.4. *AK294004*

Recent microarray analyses by Wang et al. [89] investigated changes in the lncRNA profiles in nasopharyngeal carcinoma in response to radiation in combination with curcumin treatment, following reports that curcumin (Cur) could sensitize cancer cells to radiotherapy. Among the 116 radiation-induced and Cur-reversed differentially expressed lncRNAs, six (*AF086415*, *AK095147*, *RP1-179N16.3*, *MUDENG*, *AK056098*, and *AK294004*) were identified. Further functional studies indicated that lncRNA AK294004 directly targets Cyclin D1 and exerts a negative effect. In view of the finding that Cyclin D1 is an important mediator of the cellular DNA damage response and apoptosis after radiation treatment [90], *AK294004* may be a potential lncRNA participating in radioresistance of cancer [89].

#### 4.1.5. *LncRNA-ROR*

*LncRNA-ROR* was initially identified in induced pluripotent stem cells and shown to play a key role in maintaining the properties of these cells by suppressing stress pathways such as the p53 response [91,92]. Further studies provided evidence that lncRNA-ROR serves as a suppressor of p53 in response to DNA damage [93] and contributes to cancer progression, recurrence and chemoresistance, in part, by negatively regulating p53 and miR-145 in various cancer types [92,94]. Expression of *lncRNA-ROR* is increased in several cancer types and serves as a prognosis marker including colorectal cancer [95,96,97], and silencing its expression in CRC cells enhances sensitivity to radiotherapy via negative regulation of the p53/miR-145 axis. Importantly, combination of radiotherapy with specific knockdown of *lncRNA-ROR* was shown to induce significant tumor reduction in a xenograft model [95]. Thus, *lncRNA-ROR* may present an effective potential therapeutic target in combination with radiotherapy.

#### 4.1.6. *MALAT1*

Metastasis-associated lung adenocarcinoma transcript 1 (*MALAT1*) (also termed *NEAT2* or *nuclear-enriched abundant transcript 2*) was one of the first lncRNAs identified in relation to tumorigenesis and used as a prognostic marker for development of metastatic disease and poorer survival rate in early-stage lung adenocarcinoma [98]. *MALAT1* is overexpressed in various cancers and linked to promotion of radioresistance through triggering EMT, CSC activity, and anti-apoptosis ability [99,100,101]. For instance, *MALAT1* is significantly upregulated in nasopharyngeal carcinoma (NPC) specimens or cell lines. Silencing the expression of *MALAT1* sensitizes NPC cells to radiotherapy, both in vitro and in vivo. Further investigation revealed a negative regulation loop of *MALAT1* and miR-1. SLUG, a crucial regulator of EMT, was determined as a direct target of miR-1. The function of *MALAT1* in activating EMT and CSCs via modulating the miR-1/SLUG axis supports its utility as a therapeutic target for NPC patients [101]. *MALAT1* was upregulated in esophageal squamous cell carcinoma (ESCC) and cervical cancer tissues [99,102,103]. Following radiation treatment, expression of *MALAT1* was decreased in radiosensitive but increased in both radioresistant cancer cells and clinical cases. Ectopic expression of *MALAT1* induced an increase in CKS1 in ESCC cells and decrease in miR-145 in cervical cancer cells, and which is leading to inhibition of cancer cell apoptosis after radiation treatment [99,103]. These reports collectively support a critical role of *MALAT1* in radioresistance of cancers.

#### 4.1.7. *NEAT1*

Nuclear paraspeckle assembly transcript 1 (*NEAT1*) has been identified as a transcriptional target of p53 involved in the cellular response to stress or DNA damage [104,105]. Upon activation of p53, formation of paraspeckles is stimulated in mouse and human cells. Silencing of *NEAT1* expression prevents paraspeckle formation and sensitizes preneoplastic cells to DNA damage-induced cell death, preventing chemical-induced skin tumorigenesis in mice and enhancing chemotherapy-induced cytotoxicity [105], consistent with the theory that increased DNA damage sensitizes cells to p53 reactivation therapy [106]. *NEAT1* overexpression has been reported in different types of solid tumors, such as lung cancer, esophageal cancer, CRC and HCC, whereby higher levels are associated with poor prognosis [107]. *NEAT1* targeting may therefore present a potential strategy to enhance the effectiveness of DNA-damaging chemotherapeutics and p53-reactivating molecules. Recent evidence suggested that the lncRNA *NEAT1* regulates EMT and radioresistance in NPC cells [108]. Specifically, *NEAT1* was significantly upregulated in NPC cell lines and tissues and its knockdown sensitized NPC cells to radiation in vitro. Further experiments revealed reciprocal suppression effects of *NEAT1* and miR-204. Upregulated *NEAT1* in NPC cells inhibited the targeting of miR-204 to ZEB1, an important modulator of EMT in cancer cells [10], resulting in radioresistance and EMT activation [108]. Thus, *NEAT1* is considered a potential target to enhance the effectiveness of radiotherapy.

### 4.2. LncRNAs Associated with DNA Repair

#### 4.2.1. *LINP1*

DNA repair is a complex process in cells that occurs to identify and correct damaged DNA. This process is vital for maintaining genomic integrity and crucially involved in tumorigenesis and cancer radiotherapy. Non-homologous end joining (NHEJ) is one of the major mechanisms responsible for repairing damaged DNA in cancer cells [109]. Human triple-negative breast cancer (TNBC) is an aggressive subtype that presents poor prognosis and resistance to radiotherapy [110]. Recently, an lncRNA in the non-homologous end joining (NHEJ) pathway 1 (*LINP1*) was shown to be overexpressed in TNBC [111]. Upon EGFR (epidermal growth factor receptor) activation, *LINP1* is transcriptionally upregulated via RAS-MEK-ERK signaling and AP1 (activator protein 1) transcription factors. The increased level of *LINP1* acts as a scaffold to stabilize Ku80 and DNA-PKcs interactions and coordinates the NHEJ pathway to enhance DNA repair activity. RNA expression of *LINP1* is also downregulated through interactions with miR-29 in a p53-dependent manner. Importantly, p53-mediated inhibition of LINP1 increases the sensitivity of breast tumor cells to radiotherapy [111].

#### 4.2.2. *POU6F2-AS2*

Recently, *POU6F2-AS2* was identified as a novel lncRNA involved in the DNA damage response that regulates the sensitivity of cancer cells to ionizing radiation in esophageal squamous cell carcinoma. Further experiments demonstrated that *POU6F2-AS2* interacts with YBX1 (Y-box binding protein) protein and regulates chromatin localization [112]. YBX1 has been characterized as a DNA and RNA binding protein involved in the regulation of DNA damage response, DNA repair regulation, pre-mRNA splicing and mRNA packaging. Moreover, YBX1 is highly overexpressed in multiple cancer types and may serve as a potential prognostic marker for poor outcomes and drug resistance in specific cancer types [113]. Thus, *POU6F2-AS2* may be a master regulator that participates in DNA or RNA synthesis and other processes [112].

### 4.3. LncRNAs Associated with both EMT and Radioresistance

#### 4.3.1. *TUG1*

Taurine-upregulated gene 1 (*TUG1*) was initially reported to be induced following treatment with taurine in mouse retinal cells [114]. *TUG1* has been identified as a tumor suppressor or oncogene in various cancer types [115,116,117,118,119]. Recent studies also support an important role of *TUG1* in cancer cell invasion and resistance to radiotherapy. For instance, an earlier study demonstrated a significant increase in expression of *TUG1* in high-grade bladder cancer tissues while its silencing led to suppression of cell proliferation and metastasis. *TUG1* expression was markedly elevated upon radiation treatment [120]. Notably, *TUG1* expression promoted cancer cell invasion and radioresistance via triggering EMT. Further experiments disclosed that reciprocal repression of miR-145 mediates the effects of *TUG1*. Suppression of miR-145 by *TUG1* resulted in re-expression of ZEB2 (zinc finger E-box binding homeobox 2), a master inducer of EMT downregulated by miR-145, and consequently, enhanced EMT and radioresistance [119]. Additionally, silencing of TUG1 was shown to enhance sensitivity to radiotherapy via suppression of HMGB1 (high mobility group box 1) expression [120]. HMGB1 has been identified as a chromosome-binding protein that participates in DNA repair, transcription and nucleosome packaging [121]. The data collectively indicate that *TUG1* acts as a potential regulator of radioresistance in cancer through EMT induction and DNA repair regulation.

#### 4.3.2. *HOTAIR*

Homeobox (HOX) transcript antisense RNA (*HOTAIR*) initially identified as a spliced and polyadenylated RNA participating in the promotion of carcinogenesis and cancer progression, is considered a prognosis marker for various cancer types [122,123,124]. In general, *HOTAIR* is a transacting lncRNA that interacts with Polycomb Repressive Complex 2 (PRC2) and lysine-specific demethylase 1 to negatively influence the expression of cancer-related genes [124,125]. PRC2 is a histone methyltransferase involved in epigenetic silencing during different processes, including cancer development [126]. In addition to promoting cancer progression and initiation, recent evidence indicates an important role of *HOTAIR* in radiotherapy for cancer. For instance, *HOTAIR* is upregulated in breast cancer [127], cervical cancer cells [128], CRC [129], pancreatic ductal adenocarcinoma (PDAC) and Lewis lung cancer [130,131]. Overexpression of *HOTAIR* in MDA-MB-231 causes radioresistance by promoting HOXD10 expression and activation of the PI3K/AKT-BAD signaling pathway [127]. Additionally, increased *HOTAIR* expression in cervical cancer cells is reported to enhance aggressive characteristics, such as invasion, proliferation, and radioresistance, via suppression of p21 [128]. Conversely, silencing of *HOTAIR* in CRC cells inhibits cell invasion and increases sensitivity to radiation by regulating apoptosis-related genes, such as BCL-2 and BAX [129]. In PDAC and Lewis lung cancer cells, radiation treatment was shown to repress cell viability and HOTAIR while enhancing WIF-1 (WNT inhibitory factor 1) expression. WIF-1 has been identified as an inhibitor of WNT/β-catenin signaling [132]. Silencing of HOTAIR expression promotes WIF-1 expression and inhibits the nuclear translocation of β-catenin. In contrast, upregulation of HOTAIR appears to enhance nuclear β-catenin expression via inhibiting WIF-1 expression, leading to radioresistance in both PDAC and Lewis lung cancer cells [130,131]. These findings collectively support the utility of HOTAIR as a valid therapeutic target for reversal of radioresistance in several types of cancer.

### 4.4. LncRNAs Associated with Epigenetic Regulation

#### *PARTICLE* 

*PARTICLE* (*promoter of MAT2A-antisense radiation-induced circulating lncRNA*) was recently identified as a novel lncRNA participating in the regulation of cellular response to radiotherapy [133]. *PARTICLE* is located within the *MAT2A* gene and transcribed in an antisense orientation to the forward plus strand from the *MAT2A* promoter. *MAT2A* encodes the catalytic subunit of methionine adenosyltransferase (MAT), a crucial cellular enzyme contributing to production of s-adenosylmethionine (SAM), the principal methyl donor of cells [134,135]. *PARTICLE* is upregulated in both breast cancer cell lines and cells from head-and-neck cancer (HNC) patients after radiation treatment. Radiation-induced nuclear *PARTICLE* forms a DNA-lncRNA triplex at a CpG island upstream of the *MAT2A* promoter, which provides a recruitment platform for methyltransferase and subunits of polycomb repressor complex, including G9a (euchromatic histone lysine methyltransferase 2) and SUZ12 (SUZ12 polycomb repressive complex 2 subunit), leading to transcriptional repression of MAT2A. In addition, cytosolic *PARTICLE* serves as the scaffold for MAT2A. Colocalized *PARTICLE* and *MAT2A* cytosolic transcripts are exported via exosomes in response to radiation treatment in both cancer cells and clinical samples.

MAT2A is the key enzyme catalyzing the formation of SAM, the methyl donor for transmethylation, and plays an important role in DNA repair and protein methylation. Moreover, the level of epigenetic DNA methylation is increased through radiation-induced activation of MAT2A [136]. Thus, dysregulation of PARTICLE may be an important factor influencing the outcome of radiotherapy.

### 4.5. Plasma LncRNAs Associated with Radiotherapy

#### *GAS5* 

Recently, Fayda et al. [18] evaluated the plasma levels of three lncRNAs (*GAS5*, *lincRNA-p21*, and *HOTAIR*) in the treatment response in 41 patients with HNC after chemoradiotherapy. The predictive values of these lncRNAs were investigated in patients with complete response (CR) versus those with partial response (PR)/progressive disease (PD). Data from the clinical analyses revealed significantly higher levels of post-treatment *GAS5* in patients with PR/PD, compared to patients with CR. Moreover, the pretreatment *GAS5* level was markedly increased in patients with PR/PD, compared to those with CR, in an MRI-based response evaluation. However, the levels of pre- or post-treatment *lincRNA-p21* and *HOTAIR* were not informative in terms of determining treatment response. Furthermore, *GAS5* has been reported to be down-regulated in multiple cancers and serve as a prognosis marker [137]. Thus, lncRNA *GAS5* may serve as an effective biomarker to predict treatment response in patients with HNC [18].

### 4.6. Identification of Novel LncRNAs That Potentially Participate in Resistance to Radiotherapy

5-FU-based concurrent chemoradiation is recommended as the standard treatment for colorectal cancer cells. A recent study established chemoradiation-resistant HCT116 cells to investigate the potential lncRNAs involved in treatment resistance. Following microarray expression analysis and further validation, three novel lncRNAs, *TCONS_00026506*, *ENST00000468960*, and *NR_038990*, were identified, which may serve as potential therapeutic targets for radioresistant cancer cells [138].

A recent comparison between the parental nasopharyngeal cancer (NPC) cell line, CNE-2, and radioresistant CNE-2 via next-generation deep sequencing led to the annotation of 2054 novel and 781 known lncRNAs [139]. Further validation via qRT-PCR in both established radioresistant CNE-2 and 6-10B cell lines showed that three novel lncRNAs (*n373932*, *n409627* and *n386034*) exhibit significant expression changes after radiation treatment. Further examination of the expression patterns of *n373932* and its associated gene, *SLITRK5*, in clinical specimens revealed a negative correlation between expression of *n373932* and *SLITRK5.* In view of the results, it is proposed that *n373932*, *n409627* and *n386034*, and interactions between *n373932* and *SLITRK5* are involved in radioresistance of cancer cells [139].

In another study, Zhou et al. [140] performed microarray analysis to identify changes in the lncRNA expression profiles during the time-period of development of radioresistant cells from parental hypopharyngeal squamous cell carcinoma (HSCC), FaDu, after radiation therapy. Among the consistently dysregulated lncRNAs, *TCONS_00018436* was identified as a potential lncRNA mediating resistance of HSCC cells to radiation. Further experiments demonstrated that *TCONS_00018436* exhibits anti-apoptotic activity following radiotherapy and its expression is dysregulated in recurrent HSCC clinical tissue samples [140].

## 5. Cellular Functions of LncRNAs in CSCs

Targeting of CSCs is considered a promising approach for improving radiotherapeutic outcomes and preventing tumor recurrence and metastasis. Several studies have demonstrated that the dysregulation of lncRNAs in malignant tumors is closely related to the function of CSCs. Investigations linking lncRNAs with CSCs are an increasing focus of cancer therapy. Here, we have reviewed documented studies in the literature regarding lncRNAs participating in CSC regulation.

### 5.1. LncRNAs Associated with Stemness and Self-Renew of CSCs

#### 5.1.1. *LincRNA-p21*

Recent studies have demonstrated that *lincRNA-p21* is a potent suppressor of the stem-like traits of CSCs purified from both CRC and glioma cells. *LincRNA-p21* displays anti-EMT activity and is downregulated in CRC and glioma CSCs, compared to non-CSC cancer cells. The lncRNA suppresses β-catenin signaling, leading to decreased cell viability, self-renewal, and glycolysis of CSCs. Its overexpression is reported to dramatically decrease the self-renewal capacity and tumorigenicity of CSCs in xenograft mice. Based on the findings to date, *lincRNA-p21* is considered a promising therapeutic agent against CSCs in CRC [79,141].

#### 5.1.2. *LncTCF7*

*LncTCF7* is located near the TCF7 gene that is overexpressed in HCC and NSCLC [142,143]. TCF7 plays an important role in EMT induction in both HCC and NSCLC cells. The lncRNA is strongly induced in HCC cells via the IL-6/STAT3 signaling axis and appears important for promotion of EMT by IL-6 [142,143]. LncTCF7 is overexpressed in HCC and NSCLC stem cells and shown to be critical for self-renewal while its silencing leads to suppression of the CSC fraction and stem cell-related gene expression. Notably, *lncTCF7* regulates self-renewal of HCC stem cells, as confirmed by tumor sphere formation in vitro and tumor initiating frequency in vivo. Studies to date have shown that the lncRNA recruits the SWI/SNF complex to bind the TCF7 promoter and activate its expression, and TCF7 which could activate the WNT signaling pathways to accelerate self-renewal of HCC stem cells. [142].

#### 5.1.3. *HIF2PUT*

The lncRNA *HIF2PUT* (hypoxia-inducible factor-2α promoter upstream transcript) has been identified as a promoter upstream transcript (PROMPT) of hypoxia-inducible factor-2α (HIF-2α) in CRC and osteosarcoma stem cells [144,145]. The function of PROMPTs is often associated with adjacent protein-coding transcripts [146] and HIF-2α is closely linked to stem cell properties [147]. *HIF2PUT* expression is positively correlated with HIF-2α levels in patients with osteosarcoma and CRC. Notably, combined higher expression of *HIF2PUT* and HIF-2α is predictive of poorer prognosis of patients with osteosarcoma. Knockdown of *HIF2PUT* has been shown to inhibit HIF-2α expression and CSC-related genes and properties as well as spheroid formation ability, colony formation and invasiveness in CRC cells. These studies support the potential utility of *HIF2PUT* as a novel therapeutic target for different cancers.

#### 5.1.4. *HOTAIR*

*HOTAIR* has been investigated in relation to many cancer types [122,123,124]. In breast cancer, *HOTAIR* enhances metastasis [125]. Additionally, *HOTAIR* regulates breast CSCs, and microarray profiles and functional analyses have revealed that its overexpression induces genes related to stem cell activity and EMT, including CD44, STAT3, ALDH2, ZEB1, Vimentin and SOX2, at least partially through transcriptional suppression of miR-34a [148,149]. *HOTAIR* expression is necessary for maintenance of the CSC phenotype in colon and breast cancer cell lines [149]. Furthermore, this lncRNA suppresses miR-7 expression through inhibition of HoxD10 in breast CSCs. Conversely, overexpression of miR-7 reverses EMT and decreases the CSC population in breast cancer cells via suppressing the STAT3 signaling pathway [150].

HOTAIR has also been shown to participate in the maintenance of stemness in CRC. Its silencing in CD133^+^ CRC cells leads to decreased cellular proliferation, metastasis and colony-forming properties as well as decreased Vimentin with enhanced E-cadherin expression [151]. Additionally, CD133^+^ CRC cells with low *HOTAIR* expression show decreased capacities of tumor growth and lung metastasis in xenograft mouse models [151]. Therefore, *HOTAIR* may present a potential therapeutic target against cancers.

#### 5.1.5. *Lnc34a*

The microRNA, miR-34a, is a downstream target of p53 involved in suppression of various cancer types [152]. Among its many functions, miR-34a has been shown to limit self-renewal of CSCs [153]. Recently, the lncRNA *Lnc34a*, which initiates asymmetric division of stem cells by directly targeting miR-34a and causing disruption of spatial balance, was shown to be overexpressed in CSCs of CRC [154]. *Lnc34a* recruits DNMT3A (DNA methyltransferase 3 α) via PHB2 (prohibitin 2) and HDAC1 (histone deacetylase 1) to simultaneously methylate and deacetylate the promoter region of miR-34a. The epigenetic regulation of miR-34a is independent of its upstream regulator, p53. Higher *Lnc34a* levels promote CSC self-renewal capacity and tumor growth of CRC cells in xenograft models. Moreover, *Lnc34a* is overexpressed in late-stage CRCs, leading to epigenetic silencing of miR-34a and regulation of CRC malignancy [154].

#### 5.1.6. *TUG1*

*TUG1* is cancer-related, binds to PRC1 or PRC2, and suppresses gene expression. Expression of TUG1 is closely associated with cancer progression and therapy [117,118,120,155]. Notch signaling has been shown to promote CSC self-renewal and activity and suppress differentiation [156]. Recent studies have reported that Notch-directed *TUG1* acts as an epigenetic modulator that regulates the glioma cancer stem cell population [155]. *TUG1* is upregulated in CSCs of gliomas and downregulated upon inhibition of Notch. Overexpressed *TUG1* promotes self-renewal of glioma cells by functioning as a molecular sponge for miR-145, an important CSC regulator [157], in the cytoplasm and recruiting Polycomb via YY1 binding activity to repress differentiation genes in the nucleus, such as BDNF(brain derived neurotrophic factor), NGF (nerve growth factor), and NTF3 (neurotrophin 3). *TUG1* presents another specific therapeutic target to eliminate the GSC population [155].

#### 5.1.7. *TALNEC2*

*TALNEC2* was identified as a novel E2F1-regulated lncRNA localizing to the cytosol [158]. *TALNEC2* is overexpressed in GBM from patients with poor prognosis and glioma stem cells. E2Fs serve as transcription factors involved in the regulation of cell cycle progression, in particular, G1/S transition [159,160]. Expression of *TALNEC2* is increased in synchronized cells progressing through the late G1 and early S phases. Silencing of this lncRNA in various cancer cell lines causes cell cycle arrest at the G1 phase and inhibits cellular proliferation. Further functional analyses have revealed that inhibition of *TALNEC2* triggers repression of miR-21 and miR-191, and consequently decreases the self-renewal and mesenchymal transformation of CSCs, increases radiosensitivity and prolongs the survival of xenograft mice bearing CSCs of glioma [158]. Therefore, *TALNEC2* is considered an attractive therapeutic target for GBM.

#### 5.1.8. *HOXA11-AS*

Homeobox A11 antisense (*HOXA11-AS*) is an lncRNA located near the homeobox A11 (*HOXA11*) gene that is highly expressed in several cancer types [161]. A recent study demonstrated that *HOXA11-AS* expression is correlated with poor cervical cancer prognosis. Overexpression of *HOXA11-AS* in cervical cancer cells promotes proliferation, metastasis and the CD133^+^/CD44^+^ CSC subpopulation. Conversely, its knockdown suppresses these aggressive biologic features, accompanied by decreased EMT and CSC-related genes, including NANOG, OCT4, SOX2, and β-catenin. Accordingly, *HOXA11-AS* is under investigation as a potential novel target for cervical cancer treatment [162].

#### 5.1.9. *LncRNA-Hh*

*LncRNA-Hh* was recently identified as a Notch, Hedgehog (Hh) pathway-associated lncRNA [163]. Expression of lncRNA-Hh is upregulated in TWIST-positive mammosphere cells and involved in modulation of the Hh pathway. Overexpression of *lncRNA-Hh* in breast cancer cells increases Hh signaling accompanied by elevated levels of SOX2 and OCT4 via targeting to GAS1 (growth arrest specific 1), and consequently contributes to activation of EMT, CSC maintenance and tumorigenesis of breast cancer cells. Conversely, its silencing reverses these oncogenic effects. The data suggest that the Twist-lncRNA-Hh pathway is an important link between EMT and the CSC phenotype of cancer [163].

#### 5.1.10. *Linc00617*

The lncRNA *TUNA* is required for pluripotency of mouse embryonic stem cells (mESC) [164]. *TUNA* physically binds the promoters of NANOG, SOX2, and FGF4 (fibroblast growth factor 4), and activates transcription by recruiting the protein complex including PTBP1 (polypyrimidine tract binding protein 1), hnRNP-K, and NCL (nucleolin). Recently, the human ortholog of *TUNA*, *Linc00617*, was identified on chromosome 14 [165]. *Linc00617* is overexpressed in breast cancer cell lines and cancer specimens, and closely associated with poor prognosis. Overexpression of *Linc00617* promotes metastasis of breast cancer cells and enhances EMT, accompanied by the acquisition of CSC properties. Furthermore, linc00617 has been shown to physically bind the SOX2 promoter and activate its transcription by recruiting hnRNP-K. Conversely, silencing of *linc00617* suppresses tumor progression. *Linc00617* has therefore emerged as a novel therapeutic target for aggressive breast cancer.

#### 5.1.11. *HULC*

The lncRNA, highly upregulated in liver cancer (*HULC*), is involved in HCC development and progression [166,167,168]. A recent report has shown that HULC affects the stemness of HCC cells by cooperating with *MALAT-1*, contributing to the promotion of liver cancer stem generation through binding and loading on the promoter region of telomere repeat-binding factor 2 (TRF2) to enhance telomerase activity [168]. *HULC* also regulates lipid metabolism of hepatoma via regulating miR-9-PPARA (peroxisome proliferator activated receptor α) axis [166]. The findings suggest that HULC, in combination with *MALAT1*, may contribute significantly to malignant growth of liver cancer stem cells through metabolism regulation.

#### 5.1.12. *UCA1* (*CUDR*)

Cancer-upregulated drug resistant (*CUDR*) or urothelial cancer-associated 1 (*UCA1*) is an independent prognostic biomarker highly expressing in various human tumors and involved in tumorigenesis [169,170]. Recent studies have shown that *CUDR* enhances the interactions of SET1A and phosphorylated RB1 (pRB1) in HCC, producing an activated pRB1-SET1A complex. This complex subsequently generates a high level of H3K4 trimethylation that loads on the TRF2 promoter region, causing overexpression of TRF2, which participates in the malignant transformation of HCC stem cells via inducing alterations in telomere length [169]. Concurrently, another report suggested that lncRNA CUDR functions as an oncogene via the *CUDR-HULC* and *CUDR*-β-catenin signaling pathways [171]. Mechanistically, *CUDR* upregulates HULC and β-catenin by inhibiting methylation at the HULC promoter and promoting the formation of a β-catenin promoter-enhancer chromatin loop through interactions with CTCF [171]. Furthermore, CUDR inhibits methylation of the promoter of the lncRNA *H19* by combining with Cyclin D1 to form a complex. CUDR-cyclinD1 upregulates *H19* and subsequently, TERT and C-MYC, to promote self-renewal and proliferation of HCC stem cells [172]. The results collectively suggest that *CUDR* plays a significant role in the self-renewal and proliferation of HCC stem cells through multiple signaling pathways.

#### 5.1.13. *NEAT1*

The lncRNA *NEAT1* is required for maintenance of CSCs of glioma [173,174]. NEAT1 is overexpressed in CD133^+^ human glioma primary and CD133^+^ U87 cells. In an earlier study, its knockdown in CD133^+^ glioma cells resulted in decreased colony formation, cell proliferation, metastasis and increased cell cycle arrest and apoptosis. These effects were accompanied by induction of miR-107 and inhibition of CDK6 (cyclin dependent kinase 6) protein and the microRNA let-7e. Further experiments revealed that restoration of let-7e suppresses proliferation and metastasis but promotes apoptosis in *NEAT1* knockdown CSCs of glioma, which may be attributable to repression of NRAS, a direct target of let-7e known to induce tumorigenesis and stemness [174,175]. The data support a critical role of NEAT1 in the maintenance of stemness of glioma cells via multiple pathways.

### 5.2. LncRNAs Associated with both EMT and CSCs Generation

#### 5.2.1. *LncRNA-ROR*

*LncRNA-ROR* has been identified as a modulator of cell reprogramming and pluripotency. For instance, in breast cancer, linc-ROR appears to function as a ceRNA of miR-205 to prevent degradation of ZEB2, promoting EMT and generating cells with stem cell-like properties [94]. Moreover, linc-ROR serves as a sponge for miR-145 to inhibit its suppressive effect on OCT4, SOX2 and NANOG expression [176]. *LncRNA-ROR* is also considered a key inducer of stemness transcriptional factors (OCT4, SOX2, and NANOG) and affects the CSC population in gastric cancer [177].

#### 5.2.2. *H19*

*H19* is an imprinted oncofetal lncRNA aberrantly expressed in various cancer types with multifaceted roles throughout tumorigenesis [178]. *H19* is induced by signals involving the EMT process and stemness, such as TGF-β, hypoxia, and HGF, suggesting a pivotal role in enhancing stemness of cancer cells via EMT [179]. Overexpression of lncRNA *H19* promotes metastasis, angiogenesis, and stemness in glioblastoma and cholangiocarcinoma cells through effects on EMT [180] and is associated with poor prognosis [180,181]. Furthermore, knockdown of *H19* has been shown to downregulate the stem cell-related genes SOX2, OCT4, and NANOG, as well as other CSC markers in glioblastoma and embryonic carcinoma cell lines [182,183]. These results support the utility of *H19* as a therapeutic target for cancers.

#### 5.2.3. *FOXF1-AS1*

*FOXF1-AS1* has been identified as a novel lncRNA regulating NSCLC progression [184]. Expression of *FOXF1-AS1* is downregulated in tissues of lung cancer. Overexpression of this lncRNA suppresses the migration and invasion of lung cancer cells through regulating EMT while its silencing enhances the stem-like properties of lung cancer cells. Further experiments have revealed that *FOXF1-AS1* physically associates with the PRC2 component, EZH2 (enhancer of zeste 2 polycomb repressive complex 2 subunit), and its knockdown mediates metastasis and stemness of cancer cells in the EZH2-dependent manner. The collective data suggest that *FOXF1-AS1* may serve as an effective therapeutic target for treatment of NSCLC [184].

#### 5.2.4. *MALAT1*

*MALAT1* is reported to participate in the regulation of CSCs in various cancer types [185,186]. *MALAT1* is overexpressed in CSCs of pancreatic cancer and its elimination leads to a decrease in the pancreatic CSC fraction [186]. Knockdown of *MALAT-1* has been shown to inhibit expression of SOX2, suggesting that it contributes to the CSC phenotype via SOX2 regulation. *MALAT1* has been identified as a ceRNA for both miR-200c and miR-145, both of which target SOX2 [186,187,188]. Thus, the protein appears to regulate pancreatic CSCs through the miR-200c/miR-145/SOX2 signaling axis. Additionally, loss of *MALAT-1* in the glioma stem cell line, SHG139S, is associated with suppression of stemness markers, such as SOX2 and Nestin [185].

## 6. Conclusions

Radiotherapy is one of the major treatment modes for patients with cancer and widely used for various malignant tumors [1]. Radiation treatment induces DNA damage via ionization or generation of reactive oxygen species (ROS), leading to elimination of tumor cells, but can concomitantly promote cancer cell metastasis through activation of EMT [3,8,12,13,42,44,45,46,47]. Metastasis is a major problem in cancer treatment and closely associated with the rates of morbidity and mortality [2,3,8,44]. Cancer cells with higher EMT activity have been shown to acquire stem cell-like activity [2,48]. Radiotherapy promotes the acquisition or activation of CSCs in cancer via inducing the expression of EMT-related genes [61,63]. CSCs represent a small subpopulation of tumor cells exhibiting radioresistant property within heterogeneous cancer masses. Notably, upon radiation treatment, a small number of non-stem cancer cells have been found to exhibit CSC characteristics. Radiation-induced CSC-like cells with intrinsic stem cell properties subsequently trigger relapse and metastasis of cancer (Figure 1A) [4,61,62].

Determination of CSC-related biomarkers for prediction of radiotherapy outcomes and the molecular mechanisms mediating CSCs and radioresistance remain an urgent requirement for the successful development of novel therapeutic strategies. LncRNAs have emerged as crucial players in the complex signaling network controlling the activation of CSCs and radioresistance. LncRNAs aberrantly expressed in CSCs are active participants in the major signaling pathways governing DNA damage response, DNA repair, apoptosis, and EMT [72,189].

Previous studies have suggested the crucial role of lncRNAs deregulation in cancer recurrence and prognosis [17,18]. Furthermore, advances in profiling of lncRNAs expressions in cancers have highlighted the potential roles as biomarkers in diagnosis and prognosis of the patients. Compared with protein-coding RNAs, lncRNAs are the functional molecules and which expressions are more closely associated with the real tumor status and biological function. In addition, the sensitivity and specificity of lncRNAs are higher than the conventional protein-based markers. Moreover, lncRNAs can be utilized in clinics as non-invasion and convenient biomarkers due to their presence in body fluids [190]. In the current article, several dysregulated lncRNAs involved in the regulation of radioresistance, metastasis and cancer stem cell properties, such as *ANRIL*, *TUG1*, *LOC285194*, *LncRNA-ROR*, *MALAT1*, *NEAT1*, *HOTAIR*, *POU6F2-AS2*, *GAS5*, *HIF2PUT*, *H19*, *TALNEC2*, *HOXA11-AS*, *Linc00617*, *HULC*, and *UCA1*, have been found to be associated with the outcomes of radiotherapy and act as valuable prognostic biomarkers (Table 1 and Figure 1B,C). Additionally, *HOTAIR*, *MALAT1*, *H9* and *GAS5* have been reported as prognostic markers in the plasma of cancer patients [190].

To date, two major mechanisms have emerged regarding lncRNA-mediated effects on CSC activity and radioresistance via regulation of EMT, DNA repair, apoptosis and stemness: (1) epigenetic regulation of genes, particularly via recruitment of the Polycomb repressor complex (PRC2); and (2) post-transcriptionally by acting as competing endogenous RNAs (ceRNAs) for miRNAs that target genes involved in stemness and radioresistance [100]. The potential lncRNAs influencing radioresistance through CSC generation and the molecular mechanisms involved in radioresistance and stemness are listed in Table 1. Moreover, radiotherapy has been shown to paradoxically induce metastasis of resistant cancer cells, and CSCs are proposed to have utility in predicting tumor recurrence and metastasis [2,48].

Recently, several studies utilizing large-scale genetic and molecular analyses have identified RNA-binding proteins (RBPs) as crucial regulators in genome stability after radiation treatment [191,192]. Upon DNA damage (e.g., ultraviolet and ionizing radiation), RBPs are activated to regulate DNA-damage response (DDR) involving DNA repair, cell cycle progression, and late responses involving genes regulation that influence cell fate. In addition to mRNAs, RBPs also bind lncRNAs, many of which are regulated in response to DNA damage and involved in the radioresistance. For instance, hnRNP-K RBP physically associates with *lincRNA-p21* and mediates in *trans* the transcriptional repression of a large set of genes in a p53-dependent manner [74]. Another study indicates that radiation-induced *LINP1* acts as a scaffold to stabilize Ku80 and DNA-PKcs interactions and coordinates the NHEJ pathway to enhance DNA repair activity [111]. Dysregulated *lincRNA-p21* and *LINP1* are shown to influence the radiosensitivity of cancer cells. Further studies on the lncRNAs and RBPs involved in CSCs and radioresistance should ultimately yield useful insights into the molecular mechanisms underlying radiation-induced CSC generation and cancer metastasis to facilitate the development of effective novel therapeutic strategies against cancer.

## Figures and Tables

**Table 1:** Summary of the relevant long noncoding RNAs (lncRNAs) in radioresistance, epithelial–mesenchymal transition (EMT)/metastasis and cancer stem cells (CSCs) generation in cancer.

Gene Name	Physiological Functions			Molecules and Signaling Pathways Involved	Expression Status in Cancers	Prognostic Marker of Cancer	Reference
	Radioresistance	EMT and Metastasis	CSCs Generation				
***LincRNA-p21***	●		●	WNT/β-catenin and p21	Down		[73,74,75,76,77,78,79,141]
***LOC285194***	●	●		VEGF receptor 1 and miR-211	Down	●	[80,81,82]
***ANRIL***	●	●		miR-125a, Bax, Smac and Bcl-2	Up	●	[83,84,85,86,87,88]
***AK294004***	●			Cyclin D1	Up		[89,90]
***LncRNA-ROR***	●	●	●	p53, ZEB2, Oct4, SOX2, Nanog, miR-205 and miR-145	Up	●	[91,92,93,94,95,96,97,176,177]
***MALAT1***	●	●	●	Slug, SOX2, Cks1, miR-1, miR-145 and miR-200C	Up	●	[98,99,100,101]
***NEAT1***	●	●	●	miR-204 and ZEB1	Up	●	[104,105,106,107,108,174,175]
***LINP1***	●			Ku80 and DNA-PKcs	Up		[109,110,111]
***POU6F2-AS2***	●			YBX1	ND	●	[112,113]
***TUG1***	●	●	●	miR-145, *BDNF*, *NGF*, and *NTF3*	Up	●	[114,115,116,117,118,119,120,155]
***HOTAIR***	●	●	●	p21, Bcl-2, Bax, WIF-1, HOXD10, Bcl-2, PI3K/AKT-BAD, WNT/β-catenin, CD44, STAT3, ALDH2, ZEB1, Vimentin and SOX2	Up	●	[122,123,124,125,126,127,128,129,130,131,132,148,149,150,151]
***PARTICLE***	●			MAT2A	ND		[133]
***GAS5***	●			ND	Up	●	[18,137]
**TCONS_00026506**	●			ND	ND		[138]
**ENST00000468960**	●			ND	ND		[138]
**NR_038990**	●			ND	ND		[138]
**n373932**	●			*SLITRK5*	ND		[139]
**n409627**	●			ND	ND		[139]
**n386034**	●			ND	ND		[139]
**TCONS_00018436**	●			ND	Up regulation in recurrent cancers		[140]
**lncTCF7**		●	●	TCF7	Up		[142,143]
***HIF2PUT***			●	HIF-2α	Up regulation in cancers	●	[144,145,146,147]
***Lnc34a***		●	●	miR-34a	Up		[152,153,154]
***TALNEC2***	●		●	miR-21 and miR-191	Up	●	[158,159,160]
***HOXA11-AS***		●	●	Nanog, Oct4, Sox2 and β-catenin	Up	●	[161,162]
***LncRNA-Hh***		●	●	Hh signaling, GAS1, SOX2 and Oct4	ND		[163]
***Linc00617***		●	●	Nanog, Sox2, and Fgf4	Up	●	[164,165]
***HULC***			●	TRF2, MALAT-1 and miR-9	Up	●	[166,167,168]
***UCA1 (CUDR)***		●	●	SET1A, pRB1, HULC, β-catenin, CTCF, C-Myc, cyclinD1, TERT and *H19*	Up	●	[169,170,171,172]
***H19***		●	●	SOX2, Oct4 and Nanog	Up	●	[178,179,180,181,182,183]
***FOXF1-AS1***		●	●	EZH2	Down		[184]

ND: not determined; ●: determined.
